# Enhanced Efficacy of a Codon-Optimized DNA Vaccine Encoding the Glycoprotein Precursor Gene of Lassa Virus in a Guinea Pig Disease Model When Delivered by Dermal Electroporation

**DOI:** 10.3390/vaccines1030262

**Published:** 2013-07-18

**Authors:** Kathleen A. Cashman, Kate E. Broderick, Eric R. Wilkinson, Carl I. Shaia, Todd M. Bell, Amy C. Shurtleff, Kristin W. Spik, Catherine V. Badger, Mary C. Guttieri, Niranjan Y. Sardesai, Connie S. Schmaljohn

**Affiliations:** 1 Virology Division, United States Army Medical Research Institute of Infectious Diseases, Fort Detrick, MD 21702, USA; E-Mails: eric.r.wilkinson.ctr@mail.mil (E.R.W.); kristin.w.ostrowski.civ@mail.mil (K.W.S.); catherine.v.badger.civ@mail.mil (C.V.B.); 2 Inovio Pharmaceuticals, Inc., Blue Bell, PA 19422, USA; E-Mails: kbroderick@inovio.com (K.E.B.); nsardesai@inovio.com (N.Y.S.); 3 Pathology Division, United States Army Medical Research Institute of Infectious Diseases, Fort Detrick, MD 21702, USA; E-Mails: carl.i.shaia.mil@mail.mil (C.I.S.); todd.m.bell.mil@mail.mil (T.M.B.); 4 Integrated Toxicology Division, United States Army Medical Research Institute of Infectious Diseases, Fort Detrick, MD 21702, USA; E-Mail: amy.c.shurtleff.ctr@mail.mil; 5 Office of the Chief Scientists, Headquarters, United States Army Medical Research Institute of Infectious Diseases, Fort Detrick, MD 21702, USA; E-Mail: connie.s.schmaljohn.civ@mail.mil

**Keywords:** Lassa fever, Lassa virus, arenavirus, guinea pigs, dermal electroporation, vaccination, vaccine

## Abstract

Lassa virus (LASV) causes a severe, often fatal, hemorrhagic fever endemic to West Africa. Presently, there are no FDA-licensed medical countermeasures for this disease. In a pilot study, we constructed a DNA vaccine (pLASV-GPC) that expressed the LASV glycoprotein precursor gene (GPC). This plasmid was used to vaccinate guinea pigs (GPs) using intramuscular electroporation as the delivery platform. Vaccinated GPs were protected from lethal infection (5/6) with LASV compared to the controls. However, vaccinated GPs experienced transient viremia after challenge, although lower than the mock-vaccinated controls. In a follow-on study, we developed a new device that allowed for both the vaccine and electroporation pulse to be delivered to the dermis. We also codon-optimized the GPC sequence of the vaccine to enhance expression in GPs. Together, these innovations resulted in enhanced efficacy of the vaccine. Unlike the pilot study where neutralizing titers were not detected until after virus challenge, modest neutralizing titers were detected in guinea pigs before challenge, with escalating titers detected after challenge. The vaccinated GPs were never ill and were not viremic at any timepoint. The combination of the codon-optimized vaccine and dermal electroporation delivery is a worthy candidate for further development.

## 1. Introduction

LASV, a member of the family *Arenaviridae*, is carried by persistently infected multimammate rats (*Mastomys natalensis*). Humans can become infected by inhalation of aerosolized virus shed in rodent excreta or by person-to-person or nosocomial exposure [1]. LASV is a category A pathogen on the National Institute of Allergy and Infectious Diseases list of biodefense and emerging infectious diseases and is considered a select agent by the U.S. Centers for Disease Control. LASV is endemic throughout western Africa where it is responsible for significant human morbidity and mortality. Among all hemorrhagic fever viruses, LASV is second only to dengue virus in human impact, with an estimated 100,000 to 300,000 LASV infections and 5,000 deaths occurring annually [2]. It is likely the LASV disease burden is greater than estimated as routine surveillance of endemic disease is not performed. Also, approximately 80% of people infected with LASV develop mild symptoms and may not seek medical treatment. Symptoms include fever, malaise, severe headache, and sore throat. Bleeding occurs in about one-third of patients and is a poor prognostic indicator. Pulmonary edema and respiratory distress are common in fatal cases [1]. Mortality among pregnant women is higher than other patients and can reach 30–70% [3]. Hearing loss is observed in about 30% of hospitalized patients with approximately 50% of those patients developing permanent deafness [4]. Although Lassa fever is severe and widespread, there is no evidence of repeat infection in survivors, suggesting an effective vaccine could be developed [2]. Challenges for developing a LASV vaccine include genetic diversity of virus strains [5] and an incomplete understanding of what constitutes a protective or cross-protective immune response. Additional challenges include LASV tropism for dendritic cells and macrophages, which likely interferes with the adaptive immune response, making it difficult to identify appropriate correlates of protection [6,7]. Lassa fever patients and experimentally infected nonhuman primates (NHP) develop strong antibody responses to LASV; however, those antibodies are not neutralizing antibodies and have not been found to correlate with viral clearance. Low levels of LASV neutralizing antibodies, if detected at all, are usually only present after recovery in both humans and nonhuman primates [2]. In contrast, virus clearance has been correlated with the appearance of cytotoxic T cells in LASV-infected nonhuman primates (NHP), with those animals surviving infection displaying stronger and earlier T-cell responses than those that succumbed to LASV infection [6]. Despite these correlations, it is not clear whether or not antibodies are important for protective immunity or recovery if present before LASV infection.

There are no FDA-licensed vaccines for Lassa fever, and therapy is generally limited to supportive care. Although intravenous treatment with the antiviral drug ribavirin was found to reduce mortality if given early in the course of Lassa fever, it does not prevent deafness [8]. LASV vaccine development efforts have yet to result in a clear candidate for advanced development due to ineffective protection in animal models or safety concerns. Due to the pathogenicity of LASV, as well as the requirement for handling infectious virus in high-containment laboratories, a recombinant DNA-based vaccine is an attractive alternative to conventional vaccine approaches. To date, several experimental vaccines for LASV derived from recombinant DNA have been tested in guinea pigs and NHP [9,10]. Replication competent viral-vectored candidate vaccines include recombinant vaccinia virus [11], recombinant vesicular stomatitis virus (VSV) [12], and recombinant yellow fever virus [13,14]. Replication-deficient candidate vaccines include a Venezuelan equine encephalitis virus replicon [15] and a virus-like particle (VLP) [16]. Of these candidates, the VSV replicon showed the most promise, in that four vaccinated NHP remained clinically healthy after LASV challenge, although they did develop low-level viremia [12]. Despite these promising results, safety concerns with the VSV live vector remain. With this study, we report the development of a plasmid DNA LASV vaccine delivered via either intramuscular or intradermal electroporation. Additionally, we present immunogenicity and protective efficacy of this vaccine in a lethal guinea pig challenge model. Our data indicate that DNA vaccination offers a safe and potentially effective means to induce protective immunity against LASV.

## 2. Experimental Section

### 2.1. Construction of the Non-Optimized Lassa Josiah GPC Vaccine Plasmid

The Lassa virus glycoprotein precursor (GPC) fragment was amplified by PCR using Platinum Taq High Fidelity DNA polymerase (Invitrogen) from a LASV, Josiah strain template using GPC-specific primers. The LASV GPC fragment was then cloned into the *Not*I site of expression vector pWRG7077 (Powdermed) using T4 DNA ligase (New England Biolabs), generating pLASV-GPC. The gene is flanked by a cytomegalovirus immediate early promoter (CMV IE) and a bovine growth hormone polyadenylation signal (BGH pA). The vector contains a kanamycin antibiotic-resistance gene (KAN). Resulting clones were screened for orientation and sequenced using an ABI 3100 genetic analyzer. Plasmid DNA was purified using Purelink HiPure Mega plasmid purification kit (Invitrogen).

### 2.2. Construction of Codon-Optimized Lassa Josiah GPC Vaccine Plasmid

The published sequence for LASV GPC gene (Genbank Accession number AY628203.1) was optimized by GeneArt using a proprietary algorithm. In addition to codon usage optimization, negative cis-acting sites (such as splice sites, poly (A) signals, TATA boxes, *etc.*) which may negatively influence expression, were eliminated where relevant. The GC-content of the LASV GPC gene was adjusted to prolong mRNA half-life. Codon usage was adapted to the bias of *Cavia porcellus* resulting in an improved codon adaption index (CAI) value of 0.97 (a value of 1.0 being perfect adaption) from a value of 0.68 in the original sequence. For this analysis, any CAI value above 0.9 is considered optimal to ensure robust and stable expression rates in target organisms. The optimized sequence was synthesized and subcloned into the *Not*I/*Bgl*II site of expression vector pWRG7077 (Powdermed) by GeneArt (Germany). The cloned plasmid was sent to Aldevron (Fargo, ND, USA) for scale-up and was provided as a 1 mg/mL solution.

### 2.3. Immunoprecipitation

To confirm expression, radioimmunoprecipitation assays (RIPA) of the non-optimized vaccine construct were carried out as follows. COS-7 cells at 80% confluency in T25 cell culture flasks were transfected using the FuGene 6 transfection reagent (Roche) with 5 μg of either pWRG7077 or pLASV-GPC plasmid DNA. After 24 hours, monolayers were washed, then treated with 200 μCi Promix ([^35^S]-methionine and [^35^S]-cysteine, Amersham) for 4 hours at 37 °C. Once again, monolayers were washed, the cells harvested, lysed, and supernatant collected for immunoprecipitation analysis. A negative control cell lysate was similarly prepared, excluding the Promix incubation. A volume of 5 μL of anti-LASV antibody was preincubated for 2 hours on ice with 500 μL of the negative control lysate. A volume of 10 μL/mL of 10% SDS was added to the radio-labeled lysate, then 200 μL of this mixture was added to the preincubated negative control lysate. The mixture was incubated overnight on ice. The lysate/antisera mixture was then combined with 150 μL protein G sepharose and incubated for 30 minutes on ice. Images were obtained after protein gel electrophoresis and transfer by using a Cyclone phosphorimager (Packard).

### 2.4. Electroporation Devices

#### 2.4.1. Intramuscular Electroporation Device (IMEP)

The intramuscular electroporation device was an ELGEN Twin Injector (Inovio Pharmaceuticals), which consists of an outer housing with an inner wagon carrying two standard 1 mL syringes with needles, 4 mm apart [17]. A gearing system presses the piston of the syringes when the wagon slides forward in the housing to inject DNA during the insertion. The needles subsequently serve as electrodes. The needles penetrate to a depth of approximately 1.6 cm in the targeted muscle, distributing the DNA in a columnbular fashion throughout the muscle and perfectly co-locating the electrical field with the delivered plasmid. The device operates at an applied voltage of 60 and pulses twice, each pulse of 60 ms duration. Upon completion of the electrical pulses, internal motors retract the needle electrodes from the muscle and re-house them in the enclosed wagon. The ELGEN Twin Injector is directly linked to the ELGEN 1000 pulse generator (Inovio Pharmaceuticals), which supplies power to the unit.

#### 2.4.2. ELGEN-Minimally Invasive Dermal Electroporation Device (ELGEN-MID)

The ELGEN-MID EP device allows for dermal/subcutaneous DNA delivery at a penetration depth of 5 mm using a four electrode invasive needle array. This minimally invasive intradermal device penetrates the full depth of the skin spanning the epidermis, dermis, and into the subcutaneous space. It operates at standard EP electrical parameters and standard pulse lengths (50 V, 3 pulses, 100 ms). A rectangular electrode configuration (10 mm spacing by 5 mm) and 5 mm depth penetration ensure the distribution of the electric field over a wider skin surface and depth area. Specifically, the EP applicator consists of four gold-plated stainless steel needle electrodes with trocar grinds. The electrodes administer a synchronized pulse. The ELGEN-MID device was built with attachment cord for linkage to the ELGEN 1000 pulse generator (Inovio Pharmaceuticals), which supplies power to the unit.

### 2.5. Pilot Study: IMEP with a Non-Optimized DNA Construct

Strain 13 guinea pigs were randomly divided into two groups consisting of six animals each. Group 1 received 100 µg of the mock vaccine (two sites at 50 µg per site) via IMEP. Group 2 was IMEP-vaccinated with 100 µg of plasmid pLASV-GPC, which encodes the GPC gene of LASV, Josiah strain. At each vaccination, approximately 100 mg of plasmid DNA was injected intramuscularly, followed immediately by a two-pulse delivery of 250 mA current. Three vaccinations were administered at 3-week intervals. To collect sera for analysis of virus-specific antibody titers, phlebotomy was performed on all animals before the vaccination series was initiated (pre-bleed) and just before each vaccination session (prime, boost 1, or boost 2). Viral infection was carried out under biosafety level (BSL)-4 conditions. Each animal was administered subcutaneously a single target dose of 1,000 pfu/mL of LASV (strain Josiah) in a total volume of 100 μL physiological saline. After viral infection, phlebotomy was performed at days 7, 14, 21 postinfection and at euthanasia or the study endpoint for survivors. Animals were monitored daily and assigned morbidity scores corresponding to the development of disease signs. Animals were euthanized when moribund, and surviving animals were euthanized on day 28 postinfection. Research was conducted under an IACUC approved protocol in compliance with the Animal Welfare Act, PHS Policy, and other Federal statutes and regulations relating to animals and experiments involving animals. The facility where this research was conducted is accredited by the Association for Assessment and Accreditation of Laboratory Animal Care, International and adheres to principles stated in the 8th Edition of the Guide for the Care and Use of Laboratory Animals [18].

### 2.6. Follow-On Study: IMEP *versus* ELGEN-MID with a Codon-Optimized Construct

Strain 13 guinea pigs were randomly divided into three groups consisting of eight animals each (IMEP-vaccinated group, ELGEN-MID-vaccinated group, and a virus only group) and two mock-vaccinated control groups consisting of five animals each (IMEP Mock-vaccinated group, ELGEN-MID Mock-vaccinated group). Each animal was implanted with IPTT-300 microchip transponders (BMDS) to measure body temperature. Each group received approximately 100 µg of the mock or authentic vaccine (two sites at 50 µg per site) via IMEP or ELGEN-MID. Three vaccinations were administered at 3-week intervals. To perform the vaccinations, the abdominal fur was shaved, and each animal received two administrations of either an intramuscular injection or shallow dermal injection of DNA-containing solution. For the IMEP group, animals were administered the vaccines via electroporation as described above. To collect sera for analysis of virus-specific antibody titers, phlebotomy was performed on all animals before the vaccination series was initiated (pre-bleed) and just before each vaccination session (prime, boost 1, or boost 2). Viral infection was carried out under BSL-4 conditions. Each animal was administered subcutaneously a single dose of 1,000 pfu of LASV (strain Josiah) in a total volume of 100 μL of physiological saline. After viral infection, phlebotomy was performed at days 7, 14, 21, and 28 postinfection. Animals were monitored daily and assigned morbidity scores corresponding to the development of disease signs. Animals were euthanized when moribund, and surviving animals were euthanized at day 28. Research was conducted under an IACUC approved protocol in compliance with the Animal Welfare Act, PHS Policy, and other Federal statutes and regulations relating to animals and experiments involving animals. The facility where this research was conducted is accredited by the Association for Assessment and Accreditation of Laboratory Animal Care, International and adheres to principles stated in the 8th Edition of the Guide for the Care and Use of Laboratory Animals [18].

### 2.7. Backchallenge of Codon-Optimized LASV-GPC Vaccine Survivors

Four surviving animals from the ELGEN-MID vaccinated group were held in BSL-4 for 120 days after the vaccination study endpoint to participate in a backchallenge experiment to assess if they could survive re-infection. These animals were re-challenged with a subcutaneous dose of 1,000 pfu LASV 120 days after the end of the vaccination study and monitored for weight, body temperature, and symptom development daily for 30 days postinfection.

### 2.8. Analysis of Viremia and Neutralizing Antibody Titers

Serum samples collected pre and after infection were screened for viral titers via a standard plaque assay. Vero cells, seeded in 6-well cell culture plates, were adsorbed with gentle rotation at 37 °C 5% CO_2_ with 10-fold serial dilutions of serum for 1 hour, then an overlay of 0.8% agarose in EBME fortified with 10% fetal bovine serum and 20 μg/mL gentamicin was applied to each well and allowed to solidify. Cells were incubated at 37 °C, 5% CO_2_ for 4 days, then stained with 0.001% neutral red solution in PBS. After an overnight incubation, plaques were counted and recorded. Neutralizing capabilities of antibodies in the serum were analyzed by a standard plaque reduction/neutralization test (PRNT), as follows [19]. Twofold serial dilutions of heat-inactivated guinea pig sera were pre-incubated for 1 hour at 37 °C with LASV diluted to approximately 100 pfu. Each serum dilution/virus mixture was then added to confluent Vero cells seeded in 6-well cell culture plates. The remainder of the procedure is as described above for the standard plaque assay. Plaques were counted and compared to control wells containing cells infected with LASV pre-incubated with naïve guinea pig or primate serum. Neutralizing antibody titers (PRNT_50_, PRNT_80_) values were identified as the highest dilution of serum yielding a 50% (PRNT_50_) or 80% (PRNT_80_) reduction in plaques.

### 2.9. Immunohistochemisty

Immunohistochemistry was performed on replicate tissue sections for all animals using an Envision-PO kit. A mouse monoclonal antibody against LASV virus (L52-2074-7A) was used at a dilution of 1:15,000. After deparaffinization and peroxidase blocking, tissue sections were incubated with the primary antibody at room temperature for one hour. The sections were then rinsed and incubated for 30 minutes with the peroxidase-labeled polymer (secondary antibody). The sections were rinsed, covered, and incubated with substrate chromogen solution for 5 minutes. The sections were then rinsed, stained with hematoxylin, and rinsed again. Sections were then dehydrated, cleared with Xyless and coverslipped.

## 3. Results and Discussion

### 3.1. GPC Expression from the pLASV-GPC Plasmid

**Figure 1:**
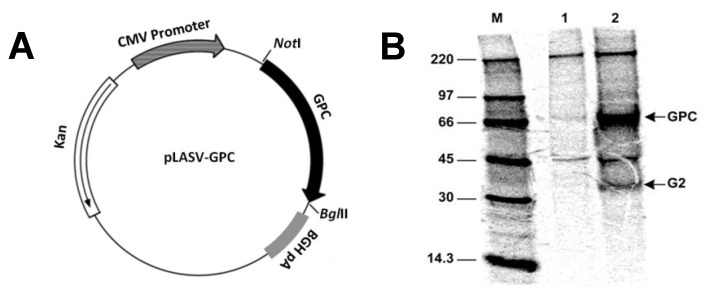
Plasmid Map and Immunoprecipitation and polyacrylamide gel electrophoresis (PAGE) of radiolabeled LASV strain Josiah glycoprotein precursor (GPC, 76 KD). (**A**) Map of pLASV-GPC cloned into the pWRG7077 vaccine plasmid. (**B**) Radioimmunoprecipitation and PAGE of LASV GPC and GP2 from COS-7 cell lysate. Expression products from COS-7 cells transfected with (Lane 1) empty vaccine plasmid pWRG7077 or (Lane 2) recombinant pLASV-GPC, and immunoprecipitated with LASV-immune guinea pig serum. The sizes of molecular weight markers M and the location of bands corresponding to GPC and GP2 are indicated.

The LASV GPC product was successfully expressed in COS-7 cells from the pLASV-GPC plasmid. Using guinea pig LASV immune serum, it was possible to immunoprecipitate GPC and GP2, which is released from GPC by post-translational cleavage through the action of a host cell subtilase SKI-1/S1P (Figure 1B, Lane 2) [20]. A plasmid map is provided as Figure 1A. Bands for GPC and GP2 do not appear in the untransfected COS cell lysate Figure 1B, Lane 1). 

### 3.2. A Non-Optimized Lassa Virus DNA Vaccine Prevents Death but not Illness in Guinea Pigs When Administered by IMEP

**Figure 2:**
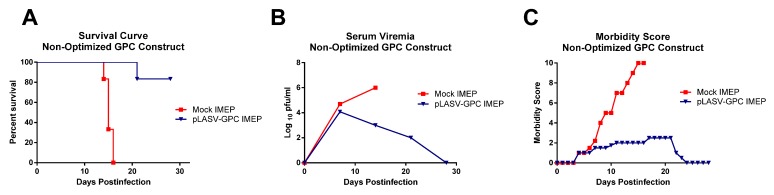
Outcomes for IMEP study using the non-optimized LASV DNA construct. (**A**) Survival curve; (**B**) Serum viremia as measured by plaque assay; (**C**) Morbidity score based on observed disease signs.

For the pilot study, we produced a candidate LASV DNA vaccine by cloning cDNA encoding the GPC gene of LASV (Josiah strain) into the plasmid vector pWRG7077 [21]. Approximately 4 weeks after the final vaccinations, the guinea pigs were challenged by SC administration of 1,000 pfu of LASV, a standard lethal challenge dose. All of the mock-vaccinated guinea pigs succumbed to LASV infection whereas all but one of the IMEP-vaccinated guinea pigs survived. The IMEP-vaccinated animal that died showed a delayed time to death as compared to controls (Figure 2A). Although the DNA vaccine prevented death in most animals, the challenged guinea pigs developed transient viremia (Figure 2B) and showed mild clinical signs of disease (Figure 2C).

Although neutralizing antibodies were not detected in vaccinated guinea pigs before challenge, they were detected on day 30 after challenge in the vaccinated animals that survived to the study endpoint, indicating that a specific immune response was elicited by the DNA vaccine (Table 1). We next sought to assess whether route of delivery might play a role in the induction of antibody responses, and in particular if we could enhance antibody responses by (a) using an optimized DNA construct and (b) by delivering the optimized LASV vaccine to the dermal tissue [21,22].

**Table 1:** Plaque-reduction neutralization test (PRNT) titers following vaccination (Day 0) and infection with LASV (Day 30) *^a^.*

*Treatment Group*	*Day 0 Postinfection*		*Day 30 Postinfection*	
	*PRNT_50_*	*PRNT_80_*	*PRNT_50_*	*PRNT_80_*
**Mock Vaccine**	None	None	-	-
**IMEP**	None	None	256	16

*^a^* Neutralizing titers are listed as the reciprocal of the dilution resulting in either 50% or 80% reduction in plaques compared to control.

### 3.3. Codon Optimization of the DNA Vaccine Enhances Its Ability to Prevent Viremia and Illness as well as Death in Guinea Pigs

In other studies, we found that optimizing codons of DNA vaccine constructs could improve both their expression and immunogenicity [23]. Subsequently, to determine if we could improve upon the protective efficacy of our DNA vaccine for LASV, we optimized the GPC construct to maximize mammalian codon availability in the guinea pig model and to remove viral elements shown to compromise expression. The optimized GPC sequence went from a codon adaption index (CAI) of 0.67 before adaption to a CAI of 0.97 after adaption, where a CAI value of 1 is considered perfect. Additional changes of note were an increase in the GC content from 43% before adaption to 60% after adaption to prolong mRNA half life and the removal of negative cis-acting sites (such as splice sites, poly A signals and TATA boxes). None of the changes in the GPC gene sequence resulted in changes at the protein level.

**Figure 3:**
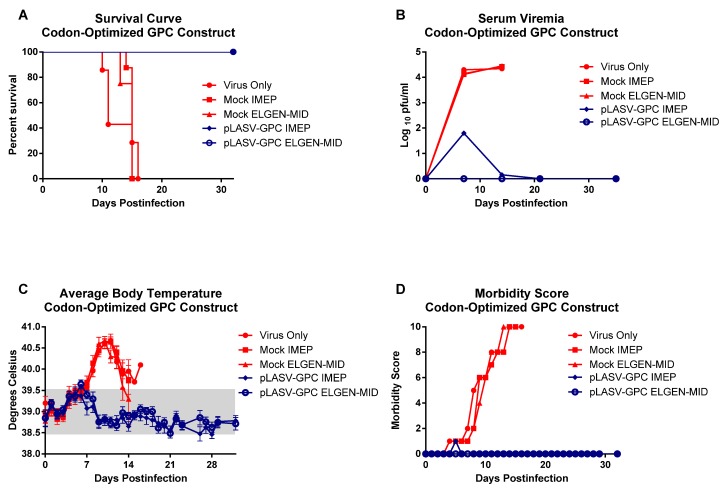
Outcomes for dermal versus muscle electroporation using the codon-optimized LASV DNA construct. (**A**) Survival curve; (**B**) Serum viremia as measured by plaque assay; (**C**) Average body temperature changes as a function of time postinfection, and (**D**) Morbidity score based on observed disease signs. The grey bar indicates the normal body temperature range for guinea pigs.

To evaluate the affect of codon-optimization alone on vaccine efficacy, we vaccinated a group of eight guinea pigs with 100 µg of DNA three times at 3-week intervals, using the same IMEP device as in the pilot study. We also vaccinated groups of guinea pigs with the optimized vaccine using a newly developed ELGEN-minimally invasive intradermal EP device (ELGEN-MID). For this study, we were able to monitor the development of fevers in control animals through the use of the IPTT-300 microchip transponders. These microchip transponders were not available for use in the pilot study. All guinea pigs mock-vaccinated with the empty plasmid or not vaccinated (virus only) became febrile, displayed signs of illness, lost weight, and succumbed to infection between days 15 and 18 postchallenge, whereas all guinea pigs vaccinated with the codon-optimized LASV DNA, regardless of the EP method used, survived challenge (Figure 3A). Unlike the pilot study in which guinea pigs vaccinated with the non-optimized LASV DNA vaccine demonstrated signs of illness, the guinea pigs vaccinated with the codon-optimized vaccine by ELGEN-MID EP did not develop any signs of illness, and remained afebrile (Figure 3B–D). We observed mild signs of disease in some of the guinea pigs that received the optimized LASV DNA vaccine by IMEP (4/8), including low fevers and slight viremias (Figure 3B,C), suggesting that dermal electroporation was more efficacious in this study. 

In addition to an improvement in outcome for vaccinated animals surviving challenge, we observed neutralizing antibodies generated as a result of vaccination before virus challenge. Unlike the pilot study in which we were not able to measure neutralizing antibodies by PRNT after three vaccinations, even at the highest serum concentration tested (1:4); detectable neutralizing antibodies were observed in all of the animals receiving the vaccine by IMEP, indicating that codon-optimization alone boosted production of neutralizing antibodies in the absence of virus (Table 2). However, the average neutralizing antibody titer for all animals in this group was just under the PRNT_50_ level for the highest serum concentration tested (1:8 dilution). Animals vaccinated via ELGEN-MID had consistently higher neutralizing antibody titers before challenge, which we believe contributed the absence of measureable viremia, fever, or other signs of disease in animals in this group. For both the IMEP and ELGEN-MID groups, at least a 20% reduction in plaque formation was maintained for dilutions out to 1:64 before challenge. After challenge, neutralizing antibody titers increased, but not significantly. We believe this is due to the vaccine protecting most of the animals from developing measureable serum viremia postchallenge, thereby mitigating the boosting affect of virus infection. The animals in the IMEP-vaccinated group that developed mild viremia and became transiently febrile (4/8) exhibited the lowest neutralizing antibody titers prechallenge (data not shown), strengthening our hypothesis that the presence of neutralizing antibodies before challenge contributed to preventing viremia in most vaccinated animals. None of the virus only (data not shown) or mock-vaccinated animals (shown in Table 2) developed measureable neutralizing antibodies before virus challenge or at euthanasia. For both the IMEP and ELGEN-MID groups, at least a 20% reduction in plaque formation was maintained for dilutions out to 1:128 at 30 days postchallenge.

**Table 2:** Plaque-reduction neutralization test (PRNT) titers following vaccination (day 0) and infection with LASV (day 30) *^a^*.

*Treatment Group*	*Day 0 Postinfection*		*Day 30 Postinfection*	
	*PRNT_50_*	*PRNT_80_*	*PRNT_50_*	*PRNT_80_*
**Mock IMEP**	None	None	-	-
**Mock ELGEN-MID**	None	None	-	-
**IMEP**	>8 ^b^	None	32	8
**ELGEN-MID**	8	None	32	8

*^a^* Neutralizing Titers are listed as the reciprocal of the dilution resulting in either 50% or 80% reduction in plaques compared to control. *^b^* The 1:8 dilution yielded an average 46% reduction in plaque formation, but two of eight animals reached the PRNT_50_ level at this dilution.

### 3.4. Surviving ELGEN-MID-Vaccinated Animals Are Free from Disease Pathology Compared to Mock-Vaccinated Controls

**Figure 4:**
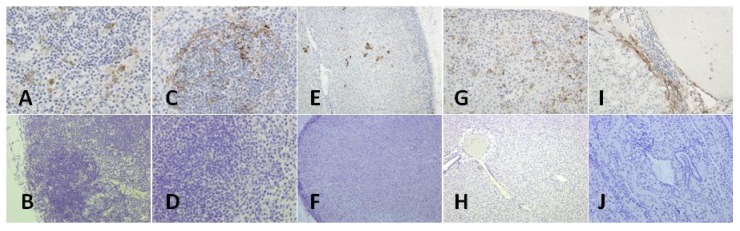
Immunohistochemistry staining for LASV antigen in selected tissues of mock-vaccinated or ELGEN-MID-vaccinated guinea pigs. (**A**) Viral antigen staining of a mock-vaccinated lymph node (40×); (**B**) lymph node of a ELGEN-MID-vaccinated animal showing lymphoid hyperplasia and a lack of viral staining (20×); (**C**) Viral antigen staining of a mock-vaccinated spleen (40×); (**D**) Splenic white pulp hyperplasia in a ELGEN-MID-vaccinated guinea pig (40×); (**E**) Viral antigen staining of a mock-vaccinated adrenal gland (10×); (**F**) A lack of viral antigen staining of a ELGEN-MID-vaccinated adrenal gland (10×); (**G**) Viral antigen staining of a mock-vaccinated liver (20×); (**H**) A lack of viral antigen staining of a ELGEN-MID-vaccinated liver (10×); (**I**) Viral antigen staining of a mock-vaccinated kidney (20×); (**J**) A lack of viral antigen staining of a ELGEN-MID-vaccinated kidney (10×).

Necropsies were performed on animals that met criteria for euthanasia or who survived to the study endpoint with the exception of four of the ELGEN-MID-vaccinated animals. These four animals will be described in the next section. Pathologic findings in virus only or mock-vaccinated animals were consistent with previous reports of the disease process in strain 13 guinea pigs [24]. There was no observed difference in lesion type or severity between the animals in the virus only group and the mock-vaccinated animals. Only mild lymphoid hyperplasia (cervical lymph node or mesenteric lymph node) and/or splenic white pulp hyperplasia was noted in some pLASV-GPC-vaccinated animals at the study endpoint. These findings are consistent with recent viral infection. None of the tissues collected from pLASV-GPC-vaccinated animals, regardless of EP method, were positive for the presence of viral antigen by immunohistochemistry at the study endpoint. Figure 4 illustrates the differences observed in antigen staining for a selection of tissues. As shown, positive LASV antigen staining was present in the lymph node, spleen, adrenal gland, liver, and kidney (Figure 4A,C,E,G,I, respectively) of a mock-vaccinated animal and absent in the corresponding tissues (Figure 4B,D,F,H,J) of a ELGEN-MID-vaccinated animal.

### 3.5. ELGEN-MID-Vaccinated Animals Survive Secondary Exposure to a Lethal Dose of LASV

**Figure 5:**
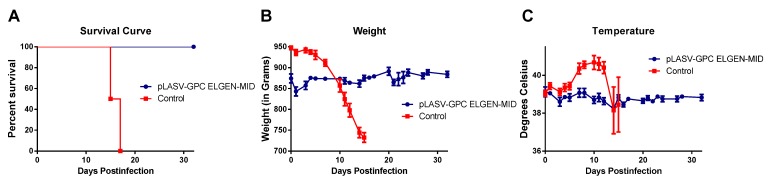
Outcome of backchallenge experiment. (**A**) Survival curve; (**B**) Average weights postchallenge; and (**C**) Average temperatures post-challenge for animals enrolled in the backchallenge experiment.

Four of the surviving ELGEN-MID vaccinated animals were kept at the end of the study in order to assess the ability of the vaccine to protect animals upon secondary exposure to virus after an extended period of time. These animals were maintained in the BSL-4 laboratory for 120 days after the end of the vaccine study, then were re-exposed to 1,000 pfu LASV by SC injection, along with four age/weight-matched control guinea pigs. These animals were observed daily for signs of disease. The vaccinated animals survived to the study endpoint (Figure 5A) and never developed signs of disease compared to the control animals, which lost weight (Figure 4B), were febrile (Figure 4C), and succumbed to disease (Figure 4A).

## 4. Conclusions

The ability to produce high levels of neutralizing antibodies before challenge is often thought of as a hallmark of a strong vaccine candidate [25,26]. While this is true for many pathogens, protective immunity against LASV in humans is thought to be primarily cell-mediated, and the role of humoral immunity and antibody production in protection is currently unclear [2,6,27,28]. While other vaccination strategies have been undertaken for LASV, to our knowledge, these studies are the first report of a non-replicating LASV vaccine to completely prevent measureable serum viremia in an animal model [12,15,29]. Our data clearly show that a plasmid encoding a codon-optimized GPC gene of LASV, when administered by dermal electroporation, can completely protect guinea pigs from viremia, illness, and death. Although low to modest neutralizing antibody titers were detected in vaccinated animals before virus challenge, we do not believe these antibodies alone account for the protection that we observed.

We demonstrated that codon optimization of the vaccine enhanced its efficacy and slightly improved its ability to elicit neutralizing antibodies. All guinea pigs receiving the non-optimized vaccine by IMEP became viremic and were mildly ill, but most survived (5/6). In contrast, guinea pigs receiving the codon-optimized vaccine by IMEP were well, experiencing only transient low-level viremia (4/8) and short-lived fever (4/8), fully resolving by the end of the study. Dermal delivery of the optimized vaccine and EP pulse was the most efficacious, with all animals surviving to the study endpoint with no viremia detected in any of the samples tested and with no observable signs of disease. Our data are consistent with what is currently known about the efficacy of different DNA vaccine delivery technologies. DNA vaccines delivered by needle injection into muscles have been shown to elicit strong cell-mediated responses but historically have not been as effective as other vaccine strategies in eliciting high levels of neutralizing antibodies in animal models [30]. In contrast, delivery of a variety of DNA vaccines to the skin of both animals and humans has been shown to elicit a more balanced humoral and cellular response and to effectively elicit neutralizing antibodies (reviewed in [21,31,32,33,34,35,36]). For example, the ability of gene gun vaccination to stimulate humoral immunity has been hypothesized to correlate with delivery of the DNA to the epidermis and specifically to the ability to target epidermal dendritic cells (Langerhans cells) [37,38,39]. Skin delivery by electroporation, similarly, and probably more efficiently, targets this same highly immunologically active site, as reflected by the improved protective efficacy observed when the LASV DNA vaccine was delivered via dermal electroporation.

Although we infer that the protective immunity observed was largely due to cell- mediated immune responses elicited by the LASV DNA vaccine delivered via electroporation, we are currently unable to provide supportive evidence for this in the guinea pig model. Unfortunately, there are few reagents available for testing the cellular immune response in guinea pigs, and the significance of correlative cytokine responses are not well defined for these animals. In ongoing studies, we are addressing this challenge by developing and incorporating gene-based array approaches to measure the guinea pig cellular responses to vaccination and virus challenge. Despite these limitations, our results clearly establish that DNA vaccination accompanied by EP is a viable strategy for inducing immunity to LASV infection and that genetic optimization of the LASV GPC sequence improves its efficacy in the guinea pig model. Further, our data provide preliminary evidence that dermal delivery by electroporation is the optimal method of vaccination with the LASV DNA vaccine. Future studies in the guinea pig model will incorporate dose and schedule refinements in order to establish the minimal protective dose for this vaccine. Additional studies in NHP are in progress, which will allow us to both obtain measurements of cell mediated immunity and to confirm our findings with the LASV DNA vaccine-dermal EP platform.
